# Phytochemicals Bridging Autophagy Induction and Alpha-Synuclein Degradation in Parkinsonism

**DOI:** 10.3390/ijms20133274

**Published:** 2019-07-03

**Authors:** Fiona Limanaqi, Francesca Biagioni, Carla Letizia Busceti, Larisa Ryskalin, Maico Polzella, Alessandro Frati, Francesco Fornai

**Affiliations:** 1 Human Anatomy, Department of Translational Research and New Technologies in Medicine and Surgery, University of Pisa, Via Roma 55, 56126 Pisa (PI), Italy; 2 I.R.C.C.S Neuromed, Via Atinense, 86077 Pozzilli (IS), Italy; 3 Aliveda Laboratories, Crespina Lorenzana, 56042 Pisa (PI), Italy

**Keywords:** curcumin, bacosides, ashwagandha, gallic/asiatic acids, resveratrol, catechins, synucleinopathy, cell-clearing pathways, metabolic syndrome

## Abstract

Among nutraceuticals, phytochemical-rich compounds represent a source of naturally-derived bioactive principles, which are extensively studied for potential beneficial effects in a variety of disorders ranging from cardiovascular and metabolic diseases to cancer and neurodegeneration. In the brain, phytochemicals produce a number of biological effects such as modulation of neurotransmitter activity, growth factor induction, antioxidant and anti-inflammatory activity, stem cell modulation/neurogenesis, regulation of mitochondrial homeostasis, and counteracting protein aggregation through modulation of protein-folding chaperones and the cell clearing systems autophagy and proteasome. In particular, the ability of phytochemicals in restoring proteostasis through autophagy induction took center stage in recent research on neurodegenerative disorders such as Parkinson’s disease (PD). Indeed, autophagy dysfunctions and α-syn aggregation represent two interdependent downstream biochemical events, which concur in the parkinsonian brain, and which are targeted by phytochemicals administration. Therefore, in the present review we discuss evidence about the autophagy-based neuroprotective effects of specific phytochemical-rich plants in experimental parkinsonism, with a special focus on their ability to counteract alpha-synuclein aggregation and toxicity. Although further studies are needed to confirm the autophagy-based effects of some phytochemicals in parkinsonism, the evidence discussed here suggests that rescuing autophagy through natural compounds may play a role in preserving dopamine (DA) neuron integrity by counteracting the aggregation, toxicity, and prion-like spreading of α-syn, which remains a hallmark of PD.

## 1. Introduction

Nutraceuticals include a broad range of naturally occurring, though different compounds such as functional foods, fortified foods, and dietary supplements which as a common signature promote human and animal health and wellness [1,2]. Among these, dietary supplements are generally identified with herbal extracts, that is, complex mixtures of phytochemicals. These latter correspond to pharmacologically active compounds, which are also named bioactive ingredients or principles. Generally, phytochemicals are classified into major categories based on their chemical structures and characteristics. These include carbohydrates, lipids, polyphenols, terpenes, steroids, alkaloids, and other nitrogen-containing compounds [3]. Phytochemicals are widely found, either singularly or in combination, in edible plants and plant products including grains, oilseeds, beans, leaf waxes, bark, roots, spices, fruits, and vegetables with varying content and composition. In the last century, phytochemicals have become increasingly popular as potential preventive and therapeutic compounds in a variety of disorders, ranging from cancer to cardiovascular, metabolic, and neurodegenerative diseases [4,5,6,7].

Natural compounds which have been mostly investigated in experimental and clinical studies for their potential benefits in brain metabolism include curcumin (*Curcuma longa*), bacosides (*Bacopa monnieri*), catechins (*Camellia sinensis*), asiatic and gallic acids (*Centella asiatica*), withanolides (*Withania somnifera, ashwagandha*), and resveratrol (*Vitis vinifera*). Clinical studies in both healthy subjects and in patients with central nervous system (CNS) disorders such as Alzheimer’s disease (AD), dementia, and amyotrophic lateral sclerosis (ALS) provided some encouraging results indicating cognitive enhancing, anti-oxidant and anti-inflammatory effects of these phytochemicals coupled with a wide margin of tolerability [8,9,10,11,12,13,14,15,16].

However, many clinical trials have not been completed yet, especially those on Parkinson’s disease (PD), and others have yielded inconclusive results. This may be due to suboptimal phytochemical dosage, timing, or formulation, which may affect phytochemical bioavailability and accumulation in the brain at necessary concentrations for producing evident therapeutic effects [9,17,18]. Strategies aimed at overcoming such a limit include the development of nanoparticle-based formulations or concomitant supplementation with natural bioavailability-enhancing compounds such as piperine [17,19]. As documented by a vast body of experimental evidence, phytochemicals from *C. longa* [9,20,21], *B. monnieri* [22,23], *C. sinensis* [24], *C. asiatica* [25], *W. somnifera, ashwagandha* [26], and *V. vinifera* [27] are indeed able to cross the blood-brain barrier thus displaying sufficient bioavailability to yield beneficial effects in the brain.

In particular, in experimental models of PD, neuroprotective effects of curcumin (*C. longa*) [28,29,30,31,32,33,34,35,36,37,38,39,40,41,42,43,44,45,46,47,48,49,50,51,52,53,54], bacosides (*B. monnieri*) [55,56,57,58,59,60,61,62,63,64,65,66,67,68], catechins of green tea (*C. sinensis*) [69,70,71,72,73,74,75,76,77,78,79,80,81], gallic and asiatic acids (*C. asiatica*) [82,83,84,85,86,87,88], withanolides (*W. somnifera*, *ashwagandha*) [89,90,91,92,93,94,95,96], and resveratrol (*V. vinifera*) [97,98,99,100,101,102,103,104,105,106,107,108,109,110,111] have been widely reported (Tables 1–6, respectively). These phytochemicals produce a number of biological effects such as modulation of dopamine (DA) metabolism and release, growth factor induction, antioxidant and anti-inflammatory activity, regulation of mitochondrial homeostasis, stem cell modulation/neurogenesis, and restoration of proteostasis through regulation of protein-folding chaperones and the cell clearing systems autophagy and proteasome [112,113,114,115,116,117,118,119,120,121,122,123,124]. As pointed by most of the past and recent discoveries in PD research, the abovementioned phytochemical-targeted processes represent key events which are altered in parkinsonism. However, when considered alone, none of these effects are expected to fully provide therapeutic efficacy in experimental parkinsonism. Indeed, PD is a multifactorial disease since different etiological (genetic and/or environmental) factors may combine to produce a chain of pathological events which tightly intermingle with each other [125,126,127,128,129,130]. These include alterations in DA metabolism and synaptic transmission, oxidative stress, mitochondrial damage, and protein aggregation. In search of convergent downstream pathways being involved in the neurobiology of PD and experimental parkinsonism, a plethora of studies indicate a key role of the cell clearing systems proteasome and autophagy [127,128,129,130,131,132,133,134,135]. In particular, autophagy is essential for DA neuronal survival being involved in the surveillance of DA release, mitochondrial homeostasis, as well as degradation of misfolded, oxidized, and aggregated proteins. The loss of autophagy in experimental models produces neurodegeneration which is reminiscent of PD [136], and autophagy dysfunctions are linked with familial PD [128]. In fact, alterations of several proteins which are encoded by PD-related genes such as alpha-synuclein (α-syn, *SNCA*), *LRRK2*, *Endophilin-A*, *PINK1*, and *Parkin*, may affect the autophagy machinery at various levels [125,128,129,130].

It is remarkable that several classes of phytochemicals converge to promote cell clearing systems, and mostly the autophagy machinery [133], either directly or by targeting common molecular pathways which are altered in parkinsonism. Thus, if one considers autophagy as a downstream common event in parkinsonism, the puzzling variety of effects induced by phytochemicals may turn to be only apparent, since different pieces can be cast together to converge towards autophagy activation. In fact, promoting autophagy contributes to regulating DA release, neuro-differentiation, and mitochondrial homeostasis, as well as counteracting oxidative/inflammatory toxicity and α-syn aggregation, which remains a hallmark of PD [127,129,133,137,138,139,140]. 

**Figure 1:**
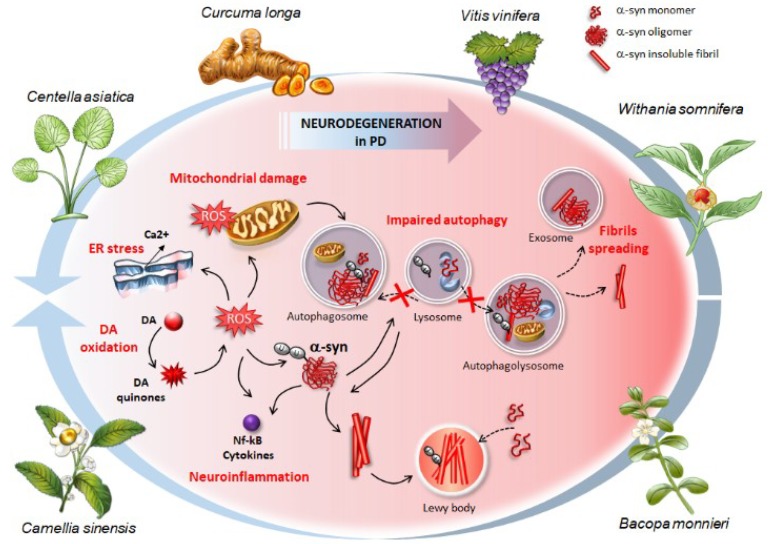
The effects of phytochemical-rich plants in counteracting the cascade (plain black arrows) of molecular events, which occur in synucleinopathies and Parkinson’s disease (PD). These include (i) oxidative stress and accumulation of Reactive Oxygen Species (ROS) arising from altered dopamine (DA) metabolism (DA oxidation), (ii) endoplasmic reticulum (ER) and mitochondrial stress, (iii) structural alterations of α-syn, formation of insoluble aggregates up to Lewy bodies where native α-syn monomers are sequestered (dashed black arrows), (iv) neuroinflammation, and (v) autophagy impairment due to either altered autophagosome biogenesis or impaired fusion between lysosomes and autophagosomes (dashed black arrows). The buildup of ubiquitinated α-syn aggregates contributes to further impairing the autophagy machinery thus fueling a vicious circle where damaged autophagy substrates accumulate due to impaired clearance and turnover. This, in turn, contributes to increasing the overall vulnerability of DA neurons and promoting the spreading of α-syn (dashed black arrows). Phytochemicals from the plants represented here confer neuroprotection by preventing or reverting (blue arrows) this pathological cascade, starting from autophagy induction to inhibition of α-syn aggregation, neuroinflammation, and oxidative stress.

It is worth mentioning that, similar to autophagy, the ubiquitin-proteasome system is affected in DA-related CNS disorders including PD [134,135,141], and a functional interplay occurs between autophagy and the proteasome at both biochemical and morphological levels [142,143]. However, here we chose to focus on the autophagy machinery for several reasons. Although both systems are seminal for DA synaptic activity and neuronal proteostasis, autophagy degrades specific substrates such as mitochondria and large protein aggregates which cannot be processed by the proteasome. Secondly, autophagy is able to compensate for proteasome dysfunctions and to rescue DA neurons from cell death which is induced by proteasome inhibitors [128,143]. Thus, in the present review we focus on autophagy as one of the final metabolic pathways through which phytochemicals restore α-syn proteostasis to confer neuroprotection (Figure 1). This might also disclose a role of autophagy dysregulations as part of a common chain of events connecting systemic disorders with alterations of the CNS, which occurs in PD. Nonetheless, the chance that phytochemicals act at the level of the proteasome system or modify its interplay with autophagy should be constantly considered.

## 2. Eukaryotic Cell Clearing Pathways: A Focus on Autophagy

Eukaryotic cell clearing pathways are grouped into two main systems, which consist of the ubiquitin-proteasome and autophagy [137,140]. The latter is further distinguished into macro-autophagy (hereafter referred to as autophagy), micro-autophagy, and chaperone-mediated autophagy [140]. In addition, other terms are used to describe the clearance of specific cell compartments, which is carried out by autophagy [144]. For instance, the removal of altered mitochondria is named “mitophagy”, which does not necessarily represent a process which is purely dedicated to removing altered mitochondria. Other examples include autophagy-dependent clearance of pathogens, ribosomes, portions of endoplasmic reticulum or synaptic vesicles which are conventionally designated as “xenophagy”, “ribophagy” or “reticulophagy”, or “vesiculophagy”, respectively [129,144]. 

Autophagy represents a phylogenetically conserved eukaryotic degradative process which plays a crucial role in cellular homeostasis [145]. A variety of cellular components encompassing proteins, lipids, sugars, nucleic acids, whole organelles or cytoplasmic compartments are sequestered into a double-membrane nascent vacuole called phagophore, which then matures to seal in a vesicle called autophagosome [146]. Autophagy engulfment may occur either as a non-selective process or involve adaptor/receptor proteins such as SQSTM1/p62 and optineurin, which shuttle ubiquitinated cargoes to the forming autophagosome [137,145,146]. The autophagosome matures through fusion with endomembrane vesicles (late endosomes and multivesicular bodies) giving birth to the amphisome. This latter eventually fuses with the lysosome, which provides acidic hydrolases needed for the breakdown of substrates. Once engulfed within the autophagolysosome, the cargo is degraded while some metabolic by-products are recycled. A complex machinery including more than 30 autophagy-related-gene (Atg) products governs the fine steps of autophagy progression, starting from the biogenesis and maturation of autophagosomes up to the fusion with lysosomes [147,148]. One of the main mechanisms negatively regulating autophagy relies on mTOR complex1 (mTORC1)-dependent phosphorylation of Atg13 and inhibition of Atg1 (ULK1 in mammals), both belonging to a molecular complex, which is seminal for the early induction of autophagy [149]. Again, conversion of Atg8 (LC3 in mammals) into LC3I, ubiquitination-like enzymatic lipidation of LC3I into LC3II isoform, and eventually the incorporation of LC3II into the phagophore membrane are critical steps for the vacuole to expand and seal, thus allowing cytoplasmic elements to be properly engulfed. In line with this, LC3 is widely employed as a marker for monitoring autophagy at the morphological, ultrastructural, and biochemical level. Nonetheless, other several autophagy proteins ranging from Atg3 to Atg7 are key in autophagy progression, since they participate in the processing and conjugation of Atg8/LC3 to the growing autophagosome’s membrane lipids [147,148]. Moreover, several pathways besides Akt/mTOR are known to modulate autophagy. For instance, autophagy occurs upon activation of 5′ AMP-activated Protein Kinase (AMPK) or following inhibition of Glycogen Synthase Kinase 3 Beta (GSK3-β) [150]. Again, activation of the transcription factor EB (TFEB) promotes autophagy induction by acting either in cooperation with or independently of mTORC1 to regulate lysosomal activation and autophagosome-lysosome fusion [151]. Likewise, activation of the NAD-dependent deacetylase Sirtuin-1 (SIRT1) promotes autophagy via de-acetylation of Atg5, Atg7, LC3 as well as of the transcription factor forkhead box O3 (FOXO3), which, in turn, controls the expression of several pro-autophagic proteins [152]. 

Autophagy modulates key cell functions ranging from cell growth and metabolism to neurotransmitter release, synaptic development and plasticity, neuro-inflammation and -immunity [125,127,129,130,132,133,135,137]. This is due to the fact that autophagy regulates the turnover of key proteins and organelles which are involved in these cell processes, and again, a mutual interplay exists between autophagy machinery and secretory/trafficking pathways, heat shock protein chaperones, apoptosis, growth factors, and inflammatory cascades. In fact, various molecules such as Rab-GTPases and SNARE proteins, heat shock proteins (HSP), caspases, reactive oxygen species (ROS), neurotrophic growth factors, pro-inflammatory cytokines/transcription factors can indirectly modulate the autophagy machinery [127,130,137,153,154,155,156,157]. Thus, it is not surprising that autophagy is commonly dysregulated in a myriad of CNS disorders where a feedback loop establishes between impaired proteostasis, synaptic alterations, and oxidative/inflammatory events. In the case of PD, this is best exemplified by the fact that DA-related oxidative/inflammatory events and α-syn aggregation may converge to impair the autophagy machinery, and, in turn, impaired autophagic clearance may fuel accumulation of toxic α-syn aggregates, synaptic alterations and neurodegeneration [127,128,129,131,158,159,160]. As we shall see in the next section, autophagy is affected in both PD patients and experimental models, and promoting autophagy counteracts α-syn aggregation and rescues DA cell death in experimental parkinsonism (Section 3). 

## 3. Autophagy Failure in Parkinson’s Disease Patients and Experimental Models

The early description of an alteration of the autophagy machinery in the brain of PD patients was carried out in the late 90s by Anglade et al. (1997) [161], who demonstrated in the Substantia Nigra pars compacta (SNpc) the concomitancy of apoptotic cells and neurons where autophagy appeared to be altered. These ultrastructural findings followed up a smoldering background, where commonalities between altered ubiquitin-dependent protein degradation and PD were already postulated by Mayer et al. [162,163]. In detail, the authors were stricken by the similarities between cell pathology developing during viral infections and neuronal inclusions observed in PD, both being cases characterized by ubiquitin-positive proteinaceous aggregates. On this basis, an altered protein degradation pathway was postulated as a common mechanism in these disorders. Indeed, alterations of autophagy machinery have been documented in the brains of patients with PD and Dementia with Lewy Bodies (DLB), featuring the occurrence of altered mitochondria within autophagy-like vacuoles, and the concomitant accumulation of LC3-II and α-syn [164,165,166,167,168]. Again, decreased levels of Atg7 along with increased levels of mTOR are detected in PD brains [169]. This occurs along with the accumulation of α-syn-filled LC3-II-positive autophagosomes, which do not co-localize with the lysosomal cathepsin D, confirming an impaired autophagy flux in PD.

The impressive insight into the genetics of PD between the end of the 90s and the first decade of 2000 led to hypotheses that autophagy failure might be a common event in PD [128]. In fact, as thoroughly reviewed elsewhere, several proteins which are coded by PARK loci-related genes play a role in autophagy machinery. Either structural changes or genetic mutations leading to a loss/gain of function of PD-related proteins such as α-syn, Synphilin, Endophilin-A, LRRK2, UCH-L1, DJ-1, Parkin, and PINK1 affect the autophagy machinery at various levels, ranging from autophagosome biogenesis to priming of aggresomes for autophagic clearance, lysosomal uptake, and degradation of substrates [125,128,129,130].

Studies on transgenic and toxin-based experimental models of parkinsonism have been seminal to confirm a key role of autophagy in the survival of DA neurons. For instance, in catecholamine-containing PC12 cell lines, the overexpression of mutant A53T human α-syn leads to cell death, which associates with impaired lysosomal degradation [170]. In detail, mutant α-syn binds to the lysosomal-associated membrane protein type 2A (LAMP-2A) to block the lysosomal uptake and inhibit both their own degradation and that of other autophagy substrates [159]. Overexpressed and mutant α-syn may also inhibit autophagy by impairing the cytosolic translocation of high mobility group box 1 (HMGB1), which blocks HMGB1-Beclin-1 binding while strengthening Beclin1-BCL2 binding [158]. As a proof of concept, when autophagy is occluded in cell lines and in cultured murine midbrain DA neurons, an accumulation of α-syn occurs [171,172,173]. Conversely, exposure to autophagy inducers such as rapamycin, a gold-standard mTORC1 inhibitor, or overexpression of Beclin-1, boosts the clearance of α-syn [158,171,173]. Again, pharmacological and genetic blockade of autophagy exacerbates DA cell loss and formation of α-syn-containing inclusions which are induced by the neurotoxic drug of abuse Methamphetamine (Meth) [143,174,175,176]. Conversely, autophagy activation is able to counteract both Meth toxicity and Meth-induced behavioral alterations [143,175,176,177]. Autophagy inhibition also exacerbates rotenone- and 6-hydroxydopamine (6-OHDA)-induced DA toxicity in vitro and in vivo [178,179], while autophagy activation protects against 6-OHDA and rotenone-induced parkinsonism [180,181]. Likewise, 1-methyl-4-phenyl-1,2,3,6-tetrahydropyridine (MPTP)-induced nigrostriatal damage in zebrafish is prevented by the overexpression of ATG5, which reduces the levels of α-syn and other indigested proteins while rescuing locomotor activity [182]. 

As demonstrated in Atg7 and Atg5-knockout (KO) mice, the presence of intracellular inclusions bearing misfolded and insoluble a-syn fibrils coupled with the degenerative and sometimes precociously lethal phenotypes, confirm the key role of constitutive autophagy in the CNS [136,183,184,185]. Remarkably, both Atg5- and Atg7-KO models fully recapitulate the severe motor impairment and neuropathology of PD patients [136,183,184,185]. In fact, the loss of autophagy in these models produces DA cell loss along with neuronal inclusions featuring protein aggregates such as α-syn, Parkin, PINK1, LRRK2, ubiquitin, and p62 [136,184]. Defective autophagy fosters protein aggregation while promoting a prion-like spreading of misfolded proteins, which is a hallmark of PD. It seems that dysfunctional autophagy due to the impaired merging of autophagosomes with endosomes and lysosomes produces an exocytotic, inter-neuronal spreading of indigested cargoes such as α-syn [186]. An impairment of the autophagy pathway is tightly intermingled with α-syn misfolding/aggregation/accumulation/spreading and, thus, with the neurobiology of PD and related “synucleinopathies” such as DLB, multisystem atrophy (MSA), pure autonomic failure (PAF), lysosomal storage diseases (LSD), and Meth abuse [127,131,187,188,189,190,191]. 

## 4. Phytochemicals: Autophagy-Based Effects and Related Potential for Alpha-Synuclein Clearance in Experimental Parkinsonism

### 4.1. Introduction to Phytochemicals and Rough Classification

Phytochemicals may be classified either on the basis of their chemical structure or the biological system in which they occur. This dual classification may produce some confusion since there is considerable overlap between the chemical types of phytochemicals and their biological distribution. Thus, as an in-depth classification of phytochemicals is far from the aim of this review, we limit to providing a brief overview of the main classes of phytochemicals which are found in the plants taken into account here. This is done in the attempt to roughly contextualize the distribution of different bioactive compounds in specific herbal compounds before moving to their biological effects focused on autophagy activation, α-syn clearance, and role in Parkinsonism. 

Within each phytochemical category, further sub-division is based on their chemical structure. For instance, polyphenols possess multiple phenolic units in their chemical structure, thus ranging from simple molecules to highly polymerized structures. Roughly, polyphenols are classified into four major classes, that is, phenolic acids, flavonoids, lignans, and stilbenes [192]. Examples of polyphenol-rich plants we chose to examine in the present review include the turmeric *C. longa* containing the polyphenol curcumin, the green tea from *C. sinensis* containing catechins and flavonoids, *C. asiatica* containing gallic acids and flavonoids, and *V. vinifera* containing resveratrol [192,193]. 

Similar to polyphenols, terpenes are classified into many categories based on the number of carbon atoms and iso­prene residues present in their structure, namely monoterpenes, sesquiterpenes, diterpenes, triterpenes, tetraterpenes, and polyterpenes [194]. All terpenes share a common 5-carbon unit named isoprene which has a branched carbon skeleton deriving from a basic 5-carbon unit named isopentane. Some triterpenes are steroidal in nature, and they are known as triterpenoid saponins. These correspond to tetracyclic or pentacyclic molecules. An example of bioactive tetracyclic triterpenoid saponins are bacosides, which represent the major class of nootropic phytochemicals found within *B. monnieri* [119]. An example of bioactive pentacyclic triterpenoid saponins are madecassosides, which are found in *C. asiatica* [193]. Steroidal tetracyclic molecules also occur as triterpenoid saponins, which are known as ergostane-type steroids. These are best exemplified by bioactive compounds known as withanolides, which consist of a steroid backbone bound to a lactone or one of its derivatives [195]. Withanolides and saponins are widely found in ashwagandha, which derives from *W. somnifera* roots [195]. Despite this rough classification, most herbal products contain both terpenoids and steroidal saponins, which indeed share many properties despite differing in their structure. 

### 4.2. Autophagy and Alpha-Synuclein Clearance as Common Effects Induced by Phytochemicals

**Figure 2:**
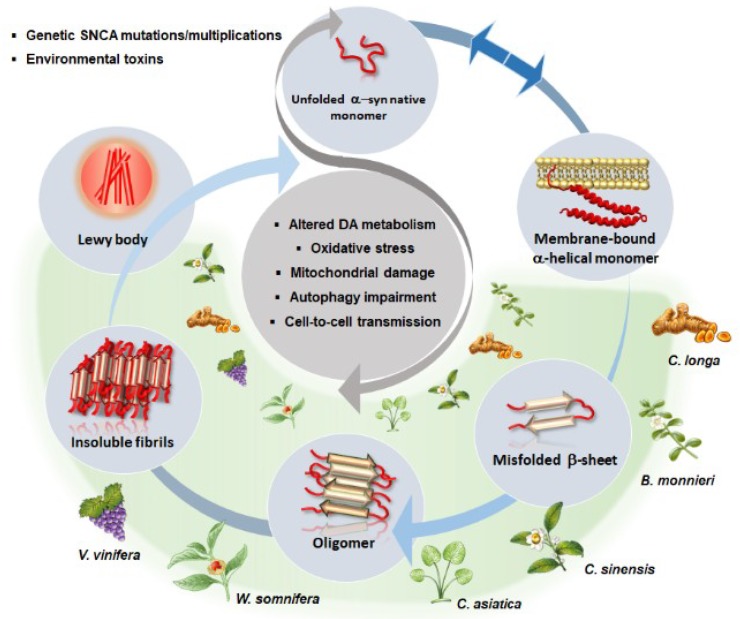
A schematic overview of the beneficial effects of phytochemical-rich plants in α-syn aggregation dynamics (light grey circles), and related molecular mechanisms (central dark grey circle) occurring in PD. In a physiological state, a dynamic equilibrium (blue arrows) exists between α-syn natively unfolded monomers and membrane-bound α-helical monomers (secondary structure). Environmental toxins or mutations/multiplications within α-syn gene (*SNCA*) favor α-syn misfolding/overexpression and drive a pathological cascade of conversion up to insoluble fibrils and Lewy body formation. This is associated with a generalized impairment of cell homeostasis consisting of altered DA metabolism and synaptic dysfunction, oxidative stress, mitochondrial damage, autophagy impairment, and cell-to-cell spreading of misfolded and aggregated α-syn conformers. Phytochemicals found within *Curcuma longa*, *Bacopa monnieri*, *Centella asiatica*, *Camellia sinensis*, *Withania somnifera* and *Vitis vinifera* are able to reverse/prevent the pathological conversion cascade of α-syn while counteracting alterations of DA neurotransmission, oxidative stress, mitochondrial damage and autophagy impairment (green shade).

All the bioactive classes above-summarized feature a remarkable overlap in their neuroprotective effects, which encompass anti-oxidant and anti-inflammatory activity, mitochondrial protection, and increased neuronal lifespan. In addition, phytochemicals exert anti-fibrillogenic effects, thus counteracting aggregation of proteins such as tau, amyloid-beta, and α-syn in the brain [196] (Figure 2). Remarkably, these phytochemicals may also act as autophagy activators, which may account for some of their beneficial effects in parkinsonism, such as counteracting α-syn aggregation. Albeit being a substrate of both autophagy and proteasome, α-syn clearance is carried out by autophagy when the proteasome is impaired, suggesting that α-syn may be a preferential substrate of autophagy [171,197]. Since α-syn dynamics are tightly bound to autophagy, which, in turn, is markedly affected in PD, in the present manuscript we focus on evidence about phytochemical-induced autophagy and α-syn clearance in experimental parkinsonism. 

#### 4.2.1. Curcumin from *Curcuma longa*

A large body of evidence converges in that curcumin may act as an autophagy inducer, which associates with various protective effects beyond the mere clearance of potentially harmful protein aggregates. For instance, curcumin promotes neurogenesis via autophagy activation [198]. In fact, in human pluripotent stem cells, curcumin upregulates neural genes along with autophagy-related genes such as Atg5, Atg8 (LC3), and Lamp1. Conversely, the inhibition of autophagy by chloroquine suppresses both autophagy and neural differentiation [198]. Furthermore, curcumin counteracts the alterations in synaptic transmission and autophagy machinery which are induced by exogenously administered misfolded proteins to cultured hippocampal neurons [199]. Again, curcumin-induced autophagy through inhibition of mTOR associates with protection from oxidative damage in several cell models [200,201]. 

The beneficial and neuroprotective effects of curcumin in PD experimental models have been widely demonstrated and thoroughly reviewed [112,113] (Table 1). The effects of chronic curcumin administration were recently evaluated in an animal model of PD induced by lipopolysaccharide (LPS) injection into the SN of rats [46]. Curcumin supplementation confers neuroprotection and attenuates motor deficits by preventing the LPS-induced neuro-inflammation and iron deposition in DA-containing neurons, and by promoting the anti-oxidant defense mechanisms along with preventing α-syn overexpression and aggregation [46], suggesting that curcumin holds potential as a candidate drug in the targeted therapy for synucleopathies. A number of studies aimed at enhancing the bioavailability and neuroprotective effects of curcumin also evaluated the effects of curcumin-based formulations against α-syn fibrillation and cytotoxicity. For instance, a nanoformulation consisting of amine-functionalized mesoporous silica nanoparticles of curcumin prevents α-syn fibrillation and subsequent cytotoxicity [202]. Another nanoformulation prepared with lactoferrin by sol-oil chemistry protects from rotenone-induced neurotoxicity in DA-containing cells through attenuation of oxidative stress along with a reduction of α-syn and tyrosine hydroxylase (TH) expression [203]. Similarly, curcumin-loaded polysorbate 80-modified cerasome nanoparticles alleviate MPTP-induced motor deficits in mice and confer neuroprotection by rescuing striatal DA levels and TH expression while promoting α-syn clearance [28]. 

A few studies focused specifically on the autophagy-based neuroprotective effects of curcumin. In detail, curcumin suppresses oxidative stress and neurotoxicity which are induced by the parkinsonian neurotoxins paraquat and atrazine through activation of autophagy in DA-containing SH-SY5Y cells [204,205]. Curcumin is able to modulate autophagy also via activation of TFEB to foster autophagy and lysosomal biogenesis in vitro and in vivo [206,207]. In keeping with this, it is remarkable that besides mTOR inhibitors, even compounds acting as TFEB activators protect from neurotoxicity in several experimental models, including parkinsonism [208]. In fact, curcumin confers protection and enhances DA cell survival by rescuing autophagy through TFEB activation in an MPTP-based cell model of PD [22]. Such an effect goes along with a reduction in α-syn levels [22], which is in line with several pieces of evidence indicating a role of curcumin-induced autophagy in counteracting α-syn aggregation and toxicity. For instance, curcumin rescues autophagy dysfunction which is induced by overexpression of mutated (A53T) α-syn in DA-containing SH-SY5Y cells, and such an effect is occluded by the autophagy inhibitor 3-MA. In turn, curcumin-induced activation of autophagy via mTOR inhibition reduces mutant α-syn accumulation to confer neuroprotection in DA cells [48]. Again, a nanoformulation containing curcumin and piperine with glyceryl monooleate nanoparticles efficiently crosses the blood-brain barrier in rotenone-induced mouse models of PD to attenuate oxidative stress and apoptosis while preventing α-syn oligomerization and fibrillation through induction of autophagy [209]. 

#### 4.2.2. Bacosides and Bacopasides from *Bacopa monnieri*

*B. monnieri* has proven potential efficacy in both in vitro and in vivo transgenic and toxin-induced experimental parkinsonism owing to its antioxidant, anti-inflammatory and neuroprotective properties [114,119] (Table 2). As a nootropic and adaptogenic compound, *B. monnieri* also acts as a DA releaser, which likely underlies its ability to ameliorate locomotor activity and cognitive functions in animal models of PD [114,119]. A few recent studies suggest that *B. monnieri* exerts its beneficial effects through autophagy activation. In fact, *B. monnieri* protects against Benzo[a]pyrene-induced oxidative stress, mitochondrial damage and cytotoxicity through autophagy induction [210]. An important standpoint in this study is that *B. monnieri* confer cytoprotection through induction of autophagy-dependent removal of damaged mitochondria, since inhibition of autophagy by Beclin-1 KO occludes its cytoprotective effects [210]. Again, bacopasides found within *B. monnieri* activate autophagy to modulate stem-cell cycle and growth [211]. 

The effects of *B. monnieri* were recently assessed in two *Caenorhabditis elegans* (*C. elegans*) PD models, namely a transgenic model overexpressing human α-syn, and a pharmacological model expressing green fluorescent protein (GFP) specifically in DA neurons treated with the selective neurotoxin 6-OHDA [65]. The study examined the effects of *B. monnieri* on α-syn aggregation in association with degeneration of DA neurons, lipids content, and longevity of the nematodes. In detail, *B. monnieri* prevents DA-neuron degeneration and increases lifespan in nematodes through reduction of α-syn aggregation and restoration of lipid content [65]. Studies investigating the effects of *B. monnieri* on α-syn aggregation and autophagy modulation specifically in parkinsonism are missing so far. However, the few available findings underlining the potential of *B. monnieri* as a possible anti-parkinsonian agent coupled with those demonstrating its pro-autophagic role, encourage further investigations on its autophagy-based neuroprotective effects in parkinsonism.

#### 4.2.3. Green Tea Catechins from *Camellia sinensis*

*C. sinensis*, the most widely used plant species for green tea, is extremely rich in polyphenols including catechins and flavonoids. Green tea catechins from *C. sinensis* show a remarkable potential in inducing autophagy [212,213]. In detail, these polyphenols modulate autophagy through various mechanisms, including TFEB, mTOR, and 5′ AMP-activated protein kinase (AMPK) [212,213,214,215]. Intriguingly, the green tea catechin epigallocatechin gallate (EGCG) was shown to activate autophagy even through direct interaction with LC3-I protein, and to foster the exposure of its pivotal Gly-120 site to other important binding partners, thus promoting the synthesis of LC3-II [216]. EGCG also activates autophagy also via a class III histone deacetylase (HDAC) [217]. Induction of autophagy by green tea polyphenols associates with various beneficial effects ranging from neuroprotection against prion protein-induced toxicity in primary neuronal cells [217] to degradation of endotoxins, anti-inflammatory activity [218] and lipid clearance [219,220]. Again, green tea catechins prevent hypoxia-induced oxidative stress and cell death by inducing autophagy [221]. Catechins can also inhibit the growth of tumor stem cells in vitro and in vivo by inducing autophagy [222,223]. Nonetheless, the autophagy-related properties of green tea depend upon the dosage used, level of stress, and the cell models employed [212]. For instance, at low-to-moderate doses, EGCG induces autophagy to prevent apoptosis and promote cell viability, while higher concentrations of EGCG may inhibit autophagy leading to apoptosis [215,224]. 

Green tea polyphenols are recognized to exert powerful neuroprotective effects in both cell-based and animal models of parkinsonism owing to their ability to counteract oxidative stress, neuroinflammation, and protein aggregation, and to promote autophagy [213,225] (Table 3). For instance, green tea polyphenols activate autophagy in DA-containing SH-SY5Y cells to confer neuroprotection from the toxic herbicide atrazine [205]. Again, EGCG protects neuronal-like, catecholamine-containing PC12 cells from oxidative-radical-stress-induced toxicity through inhibition of GSK3 pathway [226], and likely, through autophagy activation. Again, in transgenic *Drosophila* models of PD, namely mutant *LRRK2* and *Parkin*-null flies, EGCG protects from neurodegeneration and mitochondrial dysfunction through activation of AMPK, which is an upstream autophagy inducer [80]. Consistently, pharmacological or genetic activation of AMPK reproduces EGCG’s protective effects, while the loss of AMPK activity exacerbates *Parkin*-null- and mutant *LRRK*- induced DA neuronal loss and motor alterations [80]. Similar to parkin, AMPK is seminal to induce mitophagy, which occurs through AMPK-mediated phosphorylation of the autophagy initiator ATG1. This suggests that autophagy, and in particular mitophagy induction, may underlie the ability of EGCG to rescue from neurotoxicity which is induced by the enhanced LRRK2 kinase activity. 

Green tea catechins, especially EGCG, also possess a remarkable potential against α-syn aggregation and fibrillation in experimental parkinsonism [70,71,196,227]. In detail, EGCG provides neuroprotection and attenuates motor abnormalities in 6-OHDA-treated parkinsonian rats, which associates with reduced α-syn expression along with decreased mTOR, AKT, and GSK3-β levels [78]. Since inhibition of the mTOR/AKT/GSK-3β axis leads to autophagy induction, it is likely that EGCG reduces α-syn levels through autophagy-dependent protein clearance. EGCG may also prevent α-syn aggregation through modulation of the hypoxia-inducible factor (HIF)-1 signaling pathway, which in turn controls oxidative and iron homeostasis, and also autophagy-dependent mitochondrial turnover [228,229]. It is worth mentioning that EGCG modulates α-syn dynamics also through conformational [196,228] or epigenetic mechanisms [230]. In particular, EGCG interferes with an early step in the aggregation cascade by binding directly to the natively unfolded α-syn to inhibit its conversion into toxic intermediates [231]. Again, EGCG converts large, mature α-syn particles into non-toxic amorphous monomers or small diffusible oligomers displaying reduced α-syn toxicity in vitro [231]. EGCG also disaggregates α-syn fibrils by preventing the amyloid formation of α-syn tandem repeat and destabilizing α-syn fibrils into soluble amorphous aggregates [232]. In detail, EGCG appears to bind directly β-sheet-rich aggregates, thus reducing the relative concentration which is required to induce conformational changes [233]. Furthermore, EGCG modulates methylation of CpG sites within the promoter region of the α-syn gene (*SNCA*) to regulate its expression levels in the rodent brain [230].

#### 4.2.4. Gallic Acids, Asiatic Acids, and Madecassosides from *Centella asiatica*

Various in vitro and in vivo experimental studies indicate an anti-parkinsonian potential of *C. asiatica* (Table 4). Several bioactive compounds found within *C. asiatica* act as autophagy inducers, though this was mostly documented in cell-based models other than PD. For instance, madecassoside, a major bioactive component of *C. asiatica*, reduces oxidative stress and Ca2+ overload while attenuating subsequent mitochondrial damage through activation of autophagy [234]. Again, Asiatic acid triterpenoids found within *C. asiatica* downregulate stem-cell growth through inhibition of the Akt/mTOR pathway [235]. Similarly, gallic acid monophenols, which are major constituents of *C. asiatica*, act as autophagy inducers as shown by the increased abundance of LC3-II coupled with enhanced degradation of p62 [152]. Phytochemicals including gallic acids induce autophagy even through activation of SIRT1, which associates with decreased acetylation of cytoplasmic proteins. Conversely, administration of bafilomycin A1, which blocks late-step autophagy progression, occludes the beneficial effects of several phytochemicals including gallic acids [152]. 

Studies investigating autophagy-based effects of *C. asiatica* specifically in PD models are still limited so far. There is some indirect evidence based on SH-SY5Y DA cell lines. Here, Asiatic acids protect from glutamate-induced excitotoxicity by decreasing apoptosis and ROS, while stabilizing mitochondrial function through activation of the autophagy inducer SIRT1 [236]. Nonetheless, *C. asiatica* counteracts a-syn aggregation to confer neuroprotection in several PD models. In fact, *C. asiatica* inhibits α-syn aggregation from monomers, the transition of oligomers to aggregates and fosters the disintegration of the preformed fibrils [237]. Such an effect may be due to gallic acids, which prevent α-syn fibril formation while stabilizing the extended, native structure of α-syn [238]. Again, they protect from α-syn-induced toxicity by disaggregating pre-formed α-syn amyloid fibrils [239]. Interestingly, at very low concentrations and similar to what reported for EGCG, gallic acid was found to bind to and stabilize soluble, non-toxic α-syn oligomers lacking β-sheet content [239]. Again, in MPTP-treated mice and in transgenic *Drosophila* models over-expressing human α-syn, *C. asiatica* increases motor ability and it protects from neurotoxicity by reducing oxidative stress, lipid peroxidation and protein carbonyl content [85,88]. Unfortunately, these studies did not specifically asses α-syn levels or autophagy status, which underlines the need for further in vivo studies aimed at clarifying whether *C. asiatica* exerts neuroprotection through anti-α-syn and autophagy-based effects. 

#### 4.2.5. Withanolides and Withaferin from *Withania somnifera*, *ashwagandha*

Withanolides, the biologically active steroids of ashwagandha, confer neuroprotection and improve behavioral abnormalities in experimental parkinsonism, owing to their anti-oxidant, synaptic remodeling, and nerve-regenerating properties [240,241] (Table 5). Among their various biological effects, withanolides also modulate autophagy. Withaferin A, the most investigated and major constituent of ashwagandha, induces stem-cell cycle arrest and suppresses stem-cell growth through autophagy enhancement [242]. Again, ashwagandha prevents the accumulation of misfolded proteins and exerts beneficial anti-inflammatory and immunomodulatory effects, which may be due to autophagy activation. In fact, ashwagandha prevents glial activation and phosphorylation of nuclear factor kappaB (NF-κB) while inducing autophagy to reduce disease severity in SOD1(G93A) mouse model of ALS [243]. This suggests that autophagy-based effects induced by ashwagandha may be beneficial at the early stages of neurodegeneration [243]. Nonetheless, controversial results are found in the literature concerning the autophagy-related effects of ashwagandha. In fact, some studies performed in cancer cell-lines suggest that withaferin A may act as an autophagy inhibitor, or that concomitant administration of autophagy inhibitors potentiates rather than preventing the beneficial effects of withaferin A [242,244,245,246,247,248]. These controversies may be due to several factors. Firstly, similar to that reported for other phytochemicals such as green tea catechins, the effects of withaferin upon autophagy may be dose-dependent. In fact, low doses of withaferin induce autophagy as shown by the massive accumulation of LC3II puncta coupled with progressive degradation of p62 [248]. Contrariwise, higher concentrations of withaferin may stimulate endoplasmic reticulum (ER) stress to activate pro-apoptotic proteins, which may suppress autophagy-related proteins [248]. Secondly, most of the studies investigating the effects of ashwagandha on autophagy were carried out in tumor cells, where very high, toxic concentrations of Withaferin are generally employed to induce growth arrest and sensitization to apoptosis. These considerations suggest that appropriate dosing of phytochemicals is key when investigating and interpreting potential therapeutic effects. 

Despite the plethora of evidence supporting the multifold benefits of ashwagandha in experimental models of parkinsonism, only one recent study investigated the effects of withanolides specifically upon α-syn aggregation. This was carried out in stress-exposed *C. elegans* models expressing yellow fluorescent protein (YFP)-tagged α-syn [240]. In detail, Withanolide treatment produces a reduction of nearly 40% in α-syn levels compared with untreated animals. In withanolide-treated worms, such an effect goes along with lifespan extension, modulation of acetylcholine release, and enhancement of oxidative and thermal stress resistance [240]. Remarkably, all these beneficial effects depend on the insulin/insulin-like growth factor signaling (IIS) pathway, which is an upstream modulator of autophagy. Although the role of autophagy was not specifically investigated, it appears worthwhile to test in the future the effects of upon autophagy modulation and its potential contribution in parkinsonism and related synucleinopathies. 

#### 4.2.6. Resveratrol from *Vitis vinifera*

Resveratrol, a stilbene found in grapes and red wine, possesses multifold benefits including attenuation of oxidative stress, inflammation and mitochondrial impairment, modulation of stem-cell growth, neuroprotection and autophagy induction [124,249,250] (Table 6). 

Resveratrol-induced autophagy is associated with a variety of effects which may be relevant for PD. For instance, resveratrol-induced autophagy modulates embryonic stem-cell proliferation and pluripotency through AMPK/Ulk1 upregulation and mTORC1 suppression [251] and promotes neuronal differentiation of stem-cells as shown by increased expression of the neuro-progenitor markers Nestin, Musashi, and CD133 [252]. This latter effect occurs through SIRT1 activation, which besides AMPK/mTOR is one of the main mechanisms bridging resveratrol-induced beneficial effects and autophagy induction [100,248,253,254,255,256,257,258,259,260]. For instance, resveratrol-induced autophagy via SIRT1 exerts anti-inflammatory [253] and anti-bacterial activity [261], and it counteracts oxidative damage to promote cell viability [262,263]. Resveratrol-induced autophagy and mitophagy are associated with cytoprotection and anti-oxidant effects in a plethora of cell-based PD models, including exposure to the parkinsonian toxins atrazine and rotenone, and overexpression/exposure to misfolded peptides including mutant α-syn [102,104,105,107,205]. This is recapitulated in mice models of PD such as MPTP-induced parkinsonism, where resveratrol confers neuroprotection by preventing the loss of DA neurons and rescuing alterations in TH and DA levels while improving behavioral abnormalities through SIRT1-dependent autophagy activation [100]. Resveratrol also prevents α-syn aggregation and toxicity in both cell-based and animal models of parkinsonism [98,102,264,265]. For instance, in MPTP-treated rats, resveratrol reduces motor dysfunctions and alleviates the loss of DA neurons by counteracting apoptosis, neuroinflammation and α-syn aggregation [99]. Remarkably, a combined administration of resveratrol and L-DOPA also reduces the side effects of L-DOPA as well as the dosage of L-DOPA which is required to produce beneficial effects in MPTP-induced parkinsonism [99]. These effects are associated with an increased pAkt/Akt ratio [99]. Since Akt acts as a major upstream inhibitor of autophagy through activation of mTOR and/or inactivation of Beclin-1 [266], it is likely that the effects of resveratrol are bound to induction of autophagy. Indeed, specific autophagy-based effects of resveratrol in conferring neuroprotection through α-syn clearance have been widely reported. For instance, in PC12 cells overexpressing wild-type and mutated α-syn, and in rotenone-exposed SH-SY5Y cells, resveratrol enhances α-syn degradation by activating autophagy through the AMPK/SIRT1 signaling pathway [107]. Likewise, in MPTP-treated mice, the autophagy-based neuroprotective effects of resveratrol via induction via SIRT1-dependent LC3 de-acetylation occur along with a reduction in α-syn levels [100]. Contrariwise, an inhibitor of SIRT1 antagonizes the neuroprotective effects of resveratrol by reducing the autophagy-based degradation of α-syn [100]. 

**Figure 3:**
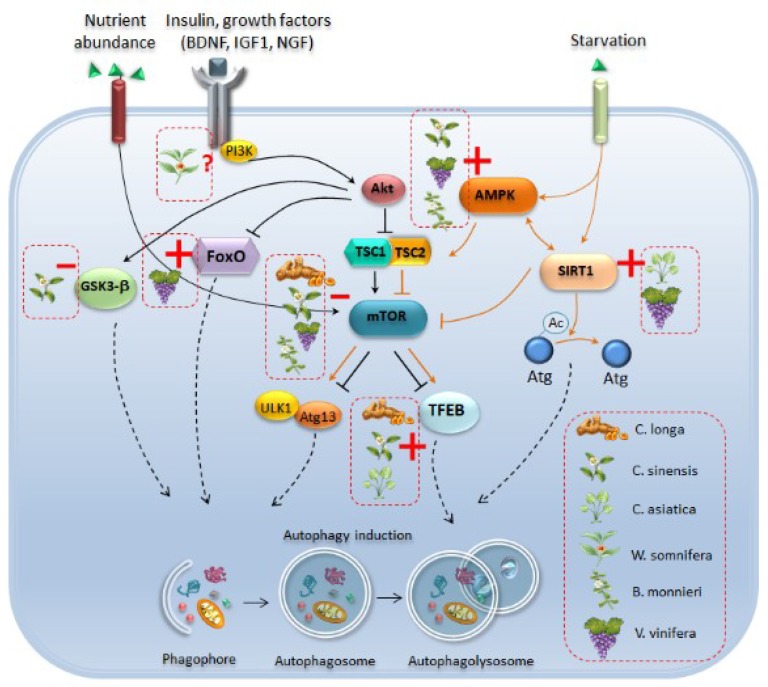
Autophagy-related molecular pathways which are targeted by phytochemical-rich plants. Phytochemicals induce autophagy by acting at several molecular levels. Curcumin (*C. longa*), catechins of green tea (*C. sinensis*), resveratrol (*V. vinifera*) and bacosides (*B. monnieri*) act as mTOR inhibitors, which leads to autophagy induction either through activation of ULK1/Atg13 or transcription factor EB (TFEB). In particular, curcumin, green tea catechins, and *C. asiatica* activate TFEB to promote its translocation to the nucleus, and the subsequent induction of autophagy-related genes. Catechins of green tea and withanolides from *W. somnifera* may also activate autophagy through inhibition of Glycogen Synthase Kinase 3 Beta (GSK-3β), while resveratrol fosters the activation of the autophagy-promoting transcription factor FoxO3. Again, green tea catechins, resveratrol and *B. monnieri* activate autophagy through enhancement of AMP-activated Protein Kinase (AMPK), which in turn is an upstream inhibitor of mTOR and an activator of Sirtuin-1 (SIRT1). Activation of SIRT1-dependent autophagy through deacetylation of Atg proteins is mainly induced by resveratrol and *C. asiatica*. Again, *W. somnifera* may also act upstream of autophagy by modulating the IGF1-Akt axis, although a role has not been confirmed yet. Plain black arrows indicate pathways which act as upstream inhibitors of autophagy while plain orange arrows indicate pathways which promote autophagy. Dashed black arrows indicate pathways converging towards autophagy machinery. Red dashed boxes indicate the specific phytochemicals which activate autophagy by acting as inhibitors (red line) or inducers (red cross) of specific autophagy-related molecules.

From these studies, it emerges that resveratrol acts quite specifically as a powerful SIRT1 activator. In fact, when compared with other phytochemicals, resveratrol induces autophagy much more potently, in a way which is reminiscent of the gold-standard autophagy activator rapamycin [152]. Such an apparently selective SIRT1-dependent mechanism recruited by resveratrol adds on the already long lists of molecules through which phytochemicals modulate autophagy, including mTOR, AMPK, TFEB, and GSK3 (Figure 3). At the same time, these considerations remark the need for further studies aimed at disclosing yet poorly explored pathways which may be involved in the autophagy-based effects of phytochemicals. In addition to these molecular findings, ultrastructural analyses seem to confirm the key role of autophagy in resveratrol-induced beneficial effects in parkinsonism. This was shown in rats with 6-OHDA-induced parkinsonism, where resveratrol exerts neuroprotective and anti-inflammatory effects [103]. Remarkably, ultrastructural analysis of DA neurons in the SN of these rats revealed that resveratrol alleviates 6-OHDA-induced subcellular alterations which are reminiscent of autophagy failure, namely accumulation of electron-dense cytoplasmic material, accumulation of vesicles resembling stagnant autophagy-like vacuoles, and mitochondrial swelling [103]. Taken together, these studies provide compelling evidence for the key role of autophagy induction in the beneficial effects of resveratrol in parkinsonism. 

## 5. Conclusions and Future Directions

The experimental evidence reviewed here converges in that phytochemicals such as curcumin, catechins of green tea, and resveratrol confer neuroprotection in experimental parkinsonism by fostering degradation of α-syn toxic species through activation of autophagy. For other phytochemical-rich plants such as *W. somnifera*, *B. monnieri*, and *C. asiatica*, the autophagy-based beneficial effects in experimental parkinsonism remain to be investigated and/or confirmed. In keeping with this, it is worth mentioning that phytochemicals may also induce autophagy indirectly. For instance, most of the compounds analyzed here, especially curcumin, EGCG, and *C. asiatica* extracts counteract the upregulation of pro-apoptotic molecules such as caspases and MAPK-p38, which may interact with Atg proteins to inhibit autophagy in favor of an apoptotic profile [130,157,267]. 

Again, neuroprotection from curcumin and *B. monnieri* extract associates with the activation of Nrf2, which in turn may induce mitophagy [268]. Curcumin and EGCG also decrease the activity of LRRK2, whose inhibition stimulates autophagy [269]. Phytochemicals are also able to restore DA levels and activity in experimental parkinsonism, and this may indirectly impact on autophagy through biochemical cascades arising from stimulation of specific DA receptors [270]. Other examples of target molecules through which phytochemicals may indirectly modulate autophagy include growth factors such as BDNF, pro-inflammatory factors, and epigenetic enzymes such as HDAC, which are all reported to have an effect upon the autophagy machinery [137,271,272].

Rescuing autophagy through natural compounds may play a role not only in preserving DA neuron integrity but also in counteracting the prion-like spreading of indigested α-syn, which is not limited to the CNS milieu but occurs even between distant cells operating in different organs [273]. In this scenario of multisystem interaction, neural mechanisms intermingle with immunological and neuroendocrine pathways to link emotional and cognitive centers of the brain with peripheral functions. In PD this is evident by the spreading of α-syn along the whole brain-gut-immune axis [273]. Since autophagy is seminal for both cellular and organ-level homeostasis, alterations of autophagy in PD are likely to underlie a much broader range of events featuring altered communication and spreading of abnormal signals between different systems. This is best exemplified by the concomitance between systemic disorders such as the metabolic syndrome and the occurrence of PD, where a failure of autophagy may represent a downstream systemic event occurring in and out the CNS. In fact, autophagy is seminal in modulating body and nutrient metabolism by acting either in peripheral organs or in the CNS by controlling hypothalamic energy expenditure, appetite, and body weight. Thus, targeting autophagy alterations through natural compounds possessing low side effects may be an advantageous strategy in targeting both CNS and systemic alterations, which occur in age-related and neurodegenerative disorders [274,275]. The beneficial effects of phytochemicals analyzed in the present review extend to several systemic diseases including metabolic syndrome, diabetes, cardiovascular disease, cancer, and chronic inflammation beyond neurodegeneration [116,274,276]. These considerations warrant additional studies aimed at dissecting and confirming the autophagy-based beneficial effects of phytochemicals in those CNS disorders such as PD, which are featured by alterations in the cell-clearing systems. In keeping with this, it would also be worth testing the effects of combined phytochemicals supplementations in PD models, which may disclose either synergistic or independent effects of single bioactive compounds. Further research is also needed to identify safe and effective strategies aimed at enhancing phytochemicals bioavailability. Again, well-designed clinical trials should be undertaken to identify the optimal dosage which can safely and effectively reproduce the beneficial effects observed in experimental models.

## Figures and Tables

**Table 1:** Neuroprotective effects of curcumin in PD models.

PD Model	Cell-Based Models	In Vivo Models
**MPTP** [28,29,30,31,32,33,34]	**Nanoparticle-loaded curcumin** in SH-SY5Y [28] ↓cytotoxicity and necrotic-like morphologic alterations ↑DA and tyrosine hydroxylase (TH) levels ↓α-syn aggregation Serum from **Curcumin-activated human mesenchymal stem cells** in PC12 cells [29] ↓apoptosis ↑neuronal differentiation, DAT and TH expression ↓pro-inflammatory cytokine release ↓nitric oxide (NO), and inducible NO synthase (iNOS) levels **Curcumin** in SH-SY5Y [30,31] ↓cytotoxicity [30,31] ↓α-syn protein and mRNA levels [30] ↑LAMP2 and LC3II and TFEB-dependent autophagy [30] ↓c-Jun, c-Jun N-Terminal Kinase (JNK), and caspase-3 [31]	**Nanoparticle-loaded** [28] **or free curcumin** [31,32,33,34] in mice ↓Parkinsonian motor symptoms [28,31,32,34] ↓loss of TH-positive neurons, depletion of DA levels and dopamine transporter (DAT)-positive fibers in the striatum [28,31,32,33,34] ↓α-syn positive Lewy Bodies [34] ↓lipid peroxidation [34] ↑antioxidant markers superoxide dismutase (SOD) and glutathione (GSH) [34] ↓JNK and caspase-related apoptotic pathways [31,32] ↑regeneration of neuroblasts in the subventricular zone (SVZ) [33] ↑growth-derived neurotrophic factor (GDNF) and transforming growth factor beta 1 (TGF-β1) levels in the SVZ [33]
**6-OHDA** [35,36,37,38,39,40]	**Curcumin** in deutocerebrum primary cells [35] ↑survival, antioxidant defense, and adhesive ability ↑Wnt/β-catenin signaling pathway **Curcumin** in MES23.5 and SH-SY5Y cells [37,38] ↓neurotoxicity ↓ROS accumulation ↓p53-mediated apoptosis ↓Nuclear Factor K Beta (NF-kβ) nuclear translocation ↑antioxidant enzyme levels and mitochondrial membrane potential (MMP) **Curcumin** in SH-SY5Y [39] ↓toxicity ↓loss of TH ↓toxic quinone formation ↓p38-Mitogen-Activated Protein Kinase (MAPK) and caspase-3	**Curcumin** in rats [35,36] ↓neurotoxicity ↓behavioral alterations ↑TH and DAT expression ↓Glial Fibrillary Acidic Protein (GFAP), Heat shock protein 70 (HSP70), and Malondialdehyde (MDA) content ↑antioxidant markers SOD and GSH ↑Wnt3/b-catenin pathway, neurotrophic growth factors (NGF) and tyrosine receptor kinase A (TrkA) expression **Curcumin** in mice [40] ↓L-DOPA-induced dyskinesia ↓Extracellular Signal-Regulated Kinase (ERK)-related AP-1 family transcription factors c-Fos, Fra, FosB, and c-Jun
**Rotenone** [41,42,43]	**Demethoxycurcumin (DMC), a derivative of curcumin**, in SH-SY5Y cells [41] ↓toxicity ↓ intracellular ROS ↓proapoptotic proteins Bax, BAD, caspase-3, -6, -8, -9 in mitochondria, and cytochrome (Cyt)-c in the cytosol ↑MMP ↑antiapoptotic markers Bcl-2, Bcl-xL, and Cyt-c in mitochondria	**Curcumin** in rats [42] ↓motor dysfunction ↑TH activity ↓GSH, Heme Oxygenase-1, and Nicotinamide Adenine Dinucleotide Phosphate Hydrogen (NADPH):quinone oxidoreductase 1 levels ↓ROS and MDA ↑Akt-Nuclear Factor Erythroid 2-Related Factor 2 (Nrf2) pathway **Curcumin** in mice [43] ↓parkinsonian behavior ↓lipid peroxidation and nitrite levels ↑antioxidant enzymes SOD, catalase (CAT), and GSH ↑Succinate Dehydrogenase (SDH) activity and mitochondrial enzyme complex activity
**Copper** [44,45]		**Curcumin** in mice [44,45] ↑locomotor activity ↑TH expression within SNc, ventral tegmental area (VTA), and dorsal striatum ↓loss of GFAP levels
**LPS** [46]		**Curcumin** in rats [46] ↓iron deposition ↓α-syn aggregation ↓pro-apoptotic markers Bax, Caspase 3, and Caspase 9 ↓inflammatory response markers GFAP, NF-kβ, tumor necrosis factor alpha (TNF-α), interleukine (IL)-1β and 1α, and iNOS ↑NADPH oxidase complex and GSH
**α-Syn** **overexpression/mutation** [47,48,49,50]	**Curcumin** in SH-SY5Y cells [47] ↓cytotoxicity induced by either extracellularly administered or intracellularly overexpressed α-syn ↓cytotoxicity of aggregated α-syn ↓ROS ↓caspase-3 activation and apoptosis **Curcumin** in SH-SY5Y cells [48] ↓A53T α-syn-induced cytotoxicity↓ cytoskeletal pathology ↓α-syn overload ↓mTOR ↑autophagy, LC3II levels and co-localization of LC3-α-syn puncta **Curcumin** in PC12 cells [49] ↓A53T α-syn-induced cytotoxicity ↓ROS, Cyt-c release, caspase-9 and -3 activation, and mitochondrial depolarization	**Curcumin** in *Drosophila* models expressing human α-syn [50] ↑lifespan and activity pattern ↓oxidative stress, apoptosis, lipid peroxidation protein carbonyl overload
***dUCH* Knockout** [51]		**Curcumin** in *Drosophila* ubiquitin carboxy-terminal hydrolase (*UCH*)-KO [51] ↓locomotor defects ↓loss of TH-positive neurons and DA levels ↓ROS
***DJ-1* Knockout** [52]		**Liposomal-formulated curcumin** in *DJ-1*-KO rats [52] ↑motor activity ↓apoptosis ↑stimulates DA neurogenesis through targeting histone deacetylase (HDAC) inhibition
***PINK1* siRNA** [53]	**Curcumin** in *PINK1* siRNA SH-SY5Y cells [53] ↓apoptosis ↑MMP and maximal respiration **Curcumin** in *PINK1* siRNA SH-SY5Y cells exposed to paraquat [53] ↔apoptosis and mitochondrial dysfunctions	
***LRRK2* mutation** [54]	**Curcumin** in *LRRK2*-transfected HEK293T cells and primary neurons treated with H2O2 [54] ↓combined cytotoxicity ↓LRRK2 kinase activity	**Curcumin** in *LRRK2*-transgenic *Drosophila* exposed to H2O2 [54] ↑survival and locomotor activity ↓loss of DA neurons ↓oxidized protein levels and LRRK2 kinase activity

Bold: The compound and the model in the tables.

**Table 2:** Neuroprotective effects of *Bacopa monnieri* in PD models.

PD Model	Cell-Based Models	In Vivo Models
**MPTP** [55,56,57,58]	***B. monnieri*** in SH-SY5Y cells [55] ↓toxicity and morphologic alterations ↑mitochondrial functions, MMP, NADH dehydrogenase, mitochondrial complex I activity ↑proteasome activity and GSH levels ↑pAkt/total Akt ratio, and activation of Nrf2	***B. monnieri*** in mice [56,57] ↓Parkinsonian motor abnormalities ↓TH-positive cell loss ↑DA and its metabolite levels ↑neurogenic genes in the SNc ↓lipid peroxidation and nitrite levels ↑antioxidant enzymes CAT, glutathione reductase and peroxidase (GR and GPx), ↓apoptotic enzymes caspase-3 and Bax ↑antiapoptotic enzyme Bcl-2 **Nanoparticle-loaded *B. monnieri*** in zebrafish [58] ↓Parkinsonian motor symptoms ↑DA and its metabolites levels ↑GSH, GPx, CAT, SOD, and mitochondrial complex-I ↓lipid peroxidation, MDA levels
**Paraquat** [55,59,60,61,62,63]	***B. monnieri*** in SH-SY5Y cells [55] ↓toxicity ↓ROS and superoxide anione levels ↑GSH and antioxidant enzymes levels ↑pAkt/total Akt ratio and Nrf2 activation ***B. monnieri*** in PC12 cells [60] ↓toxicity ↑TH levels ↓ROS, superoxide anion, MMP ↑antioxidant systems glutamylcysteine synthetase (GCS) and thioredoxin1 (Trx1) levels ↓activation of Akt and HSP90	***B. monnieri*** in *Drosophila* [59,63] ↓oxidative stress, mitochondrial dysfunctions, and lethality [59,63] ↑survival and locomotor activity [63] ↓MDA, ROS and H2O2 levels [59,63] ↓apoptosis-associated genes and proteins JNK, caspase-3 [63] ↑SDH, mitochondrial complex I-III and II-III enzymes, CAT, and ATP [59,63] ***B. monnieri*** in mice [61,62] ↓behavioral alterations in mice ↓oxidative stress, mitochondrial dysfunctions, and neurotransmitter alterations ↓ROS, MDA and H2O2 levels ↑SDH and mitochondrial complex enzymes activities ↑cholinergic enzymes activity and striatal DA levels
**6-OHDA** [64,65]		***B. monnieri*** in rats ↓behavioral alterations ↓lipid peroxidation ↑GSH content, and the amount and activities of the antioxidant GPx, GST, SOD, and CAT enzymes [64]. ***B. monnieri*** in *C. elegans* ↓loss of GFP-tagged DA neurons [65]
**Rotenone** [66,67]	***B. monnieri*** in N27 DA-cells [66] ↓toxicity ↓ROS and H2O2 levels ↑GSH levels	***B. monnieri*** in flies and mice [66,67] ↓toxicity and motor alterations ↑cholinergic enzymes activity and striatal DA levels ↓lipid peroxidation, MDA and H2O2 levels, protein carbonyl content ↑GSH, SOD and CAT content
***PINK1*-KO** [68]		***B. monnieri*** in *PINK1*-KO flies [68] ↑climbing ability
**α-Syn Overexpression** [65]		***B. monnieri*** in *C. elegans* transgenic models overexpressing human α-syn [65] ↓α-syn aggregation ↑lipid content

Bold: The plants and models in the table.

**Table 3:** Neuroprotective effects of *Camellia sinensis* in PD models.

PD Model	Cell-Based Models	In Vivo Models
**MPTP** [69,70,71,72,73]	**EGCG** in PC12 cells [69] ↓cytotoxicity ↓ROS production ↑antioxidant enzymes SOD1 and GPx ↑SIRT1/ Peroxisome Proliferator-Activated Receptor Gamma Coactivator 1-alpha (PGC-1α) pathway	**EGCG** in mice [70,71,72,73] ↓motor abnormalities [72,73] ↓loss of TH-positive neurons [70,71,73] ↑striatal DA levels, TH amount and activity [70,71,72] ↓α-syn accumulation [70,71] ↑Bcl-2 ↓Bax [70,71] ↑Protein Kinase C alpha (PKC-α) overexpression [70,71] ↓oxidative stress and protein carbonyl content [72] ↑ iron-export protein ferroportin [72] ↓the ratio of CD3^+^CD4^+^ to CD3^+^CD8^+^ T lymphocytes in the peripheral blood [73] ↓TNF-α and IL-6 in the serum [73]
***DJ-1*-KO/*Parkin*-KO + Paraquat** [74,75]		**Catechins (EGCG and propyl gallate, PG)** in paraquat-exposed *DJ-1*-KO or *Parkin*-KO *Drosophila* [74,75] ↑life-span and locomotor activity ↓degeneration of TH-positive neurons ↓lipid peroxidation
**6-OHDA** [76,77,78]	**EGCG** in PC12 and SH-SY5Y cells [76] ↓toxicity ↓Nf-kβ nuclear translocation and binding activity	***C. sinensis* extracts and catechins** in rats [77] ↓behavioral alterations ↑TH- and cyclooxygenase (COX)-2 immunopositivity ↑DA and its metabolites levels in the striatum ↓lipid peroxidation, nitrite levels, and iNOS immunopositivity **EGCG** in rats [78] ↓motor alterations and apoptosis in the SN ↓α-syn, mTOR, AKT, and GSK3β levels
**Rotenone** [79]	**EGCG** in RGC-5 [79] ↓toxicity ↓lipid peroxidation ↓MAPK, c-Jun, JNK, and p38	
***Parkin*-KO and/or** ***LRRK2* mutation** [80]		**EGCG** in *Parkin*-null or *LRRK2*-mutated *Drosophila* [80] ↑climbing scores ↓loss of DA neurons ↑mitochondrial integrity ↑activation of AMPK
**α-Syn overexpression** [81]		**EGCG** in *Drosophila* expressing human α-syn in the brain [81] ↑climbing ability ↓apoptosis and lipid peroxidation

Bold: The names of plant/bioactive compounds and models.

**Table 4:** Neuroprotective effects of *Centella asiatica* in PD models.

PD Model	Cell-Based Models	In Vivo Models
**MPTP** [82,83,84,85]		***C. asiatica*** in mice [82,83] ↓motor abnormalities [82,83] ↑DA levels, DAT and vesicular monoamine transporter type 2 (VMAT2) in the SN and striatum [82] ↑Brain-Derived and Vascular-Endothelial Growth Factors (BDNF, VEGF), GDNF, and TrKB [82] ↓MAPK-P38 related activation of JNK and ERK [82] ↑SOD, CAT, GPx, and GSH [83] ↓lipid peroxidation [83] ***C. asiatica*** in rats [84,85] ↓motor abnormalities [84] ↑DA and its metabolite levels [84] ↓lipid peroxidation, MDA, and protein carbonyl content [84,85] ↑GSH, Bcl-2/Bax ratio, BDNF [84] ↑SOD, CAT, GPx, and GSH [85]
**Rotenone** [86,87]	***C. asiatica*** in SH-SY5Y cells [86] ↓cytotoxicity, ROS, apoptosis, and DNA damage ↑MPP, Bcl-2 ↓Bax, Cyt-c, caspases-3, -6, -8, and -9	***C. asiatica*** in rats [87] ↓motor deficits ↓loss of TH-immunopositivity in the SN and striatum ↓ lipid peroxidation, MDA levels ↑mitochondrial complex I activity, SOD, and CAT
**α-syn overexpression** [88]		***C. asiatica*** in *Drosophila* expressing human α-syn in the brain [88] ↑climbing ability and activity pattern ↓lipid peroxidation, MDA, and protein carbonyl content ↑GSH
***PINK1*-KO** [68]		***C. asiatica*** in *PINK1*-KO *Drosophila* [68] ↑climbing ability

Bold: The names of plants and models.

**Table 5:** Neuroprotective effects of *W. somnifera* (ashwagandha) in PD models.

PD Model	In Vivo Models
**MPTP** [83,89,90,91]	**Ashwagandha** in mice [83,89,90,91] ↓Parkinsonian motor abnormalities [83,89,90,91] ↑DA and its metabolite levels [89,90,91] ↑GSH, GPx, SOD, and CAT [83,89,90,91] ↓lipid peroxidation and thiobarbituric acid reactive substance (TBARS) [83,89,90,91]
**Maneb-Paraquat** [92,93]	**Ashwagandha** in mice [92,93] ↓behavioral alterations and TH loss ↓ROS, lipid peroxidation, iNOS, Bax, GFAP ↑Bcl-2, CAT
**6-OHDA** [94]	**Ashwagandha** in rats [94] ↓behavioral alterations ↑TH expression, DA and its metabolite levels, DA D2 receptor binding ↓lipid peroxidation ↑GSH, GPx, GR, GST, SOD, and CAT
**Rotenone** [95]	**Ashwagandha** in *Drosophila* [95] ↓toxicity and motor alterations ↑striatal DA levels ↓ROS, lipid peroxidation, and H2O4 ↑GSH, GST, SOD, and CAT ↑SDH, mitochondrial complex-I-III and complex-II-III
***LRRK2* mutation** [96]	**Ashwagandha** in adult *Drosophila* [96] ↑lifespan, locomotor activity, muscle electrophysiological response to stimuli ↓mitochondria degeneration
***PINK1*-KO** [68]	**Ashwagandha** in *PINK1*-KO *Drosophila* [68] ↑climbing ability

Bold: The compunds and models.

**Table 6:** Neuroprotective effects of resveratrol in PD models.

PD Model	Cell-Based Models	In Vivo Models
**MPTP** [97,98,99,100,101]	**Resveratrol** in SH-SY5Y cells [97] ↓cytotoxicity and apoptosis ↓α-syn mRNA levels ↓metastasis-associated lung adenocarcinoma transcript 1 (MALAT1) and miR-129 expression	**Resveratrol** in mice [97,98,99,100] ↓Parkinsonian motor symptoms [98,99,100] ↓loss of TH-positive neurons and striatal DA depletion [97,98,99,100] ↓α-syn levels [97,99,100] ↓apoptosis, Bax and Caspase 3 [97,99] ↓MALAT1 and miR-129 expression [97] ↓proinflammatory cytokine IL-1β and GFAP [99] ↑pAkt/Akt ratio [99] ↓p62 levels [100] ↑SIRT1 and autophagy [100] **Resveratrol** in *Drosophila* [101] ↓behavioral deficits and brain histopathology ↑survival rate and life-span ↓H2O2 and nitric oxide (NO) ↑GST and CAT
**6-OHDA** [102,103]	**Resveratrol** in SK-N-BE cells [102] ↓cytotoxicity ↓ROS ↑SIRT1-dependent autophagy	**Resveratrol** in rats [103] ↓behavioral alterations induced by apomorphine-and **6-OHDA** ↓ultrastructural alterations: chromatin condensation and clumping, mitochondrial tumefaction, and vacuolization ↓COX-2 and TNF-α
**Rotenone** [104,105,106,107,108,109]	**Resveratrol** in SH-SY5Y and PC12 cells [104,105,106,107] ↓cytotoxicity and mitochondrial damage [104,105,106,107] ↓ROS and apoptosis [104,105,106] ↓histone-associated DNA fragmentation [107] ↓α-syn aggregation [107] ↓cleaved Poly ADP-ribose Polymerase (PARP) [107] ↑p-ERK1/2/ERK1/2 ratio [104,105] ↑autophagy [104,105,106,107] ↑Heme Oxygenase-1-dependent autophagy [104,105] ↑SIRT1 pathway and autophagy [106,107]	**Nanoparticle-loaded** [108] **and free resveratrol** [109] in rats ↓Parkinsonian motor dysfunction [108,109] and nigral histopathology [108] ↓striatal DA depletion [109] ↓lipid peroxidation, MDA [108] ↓ER stress markers CHOP and GRP78 [109] ↓caspase 3 activity, IL-1β level, protein carbonyl content [109] ↑SDH, citrate synthase, aconitase, and mitochondrial complex I activity [108] ↑antioxidant GSH, CAT, GPx [108,109] ↑Nrf2 DNA-binding activity [109]
**α-Syn mutation** [102,110]	**Resveratrol** in SK-N-BE cells [102] ↓A30P α-syn-induced cytotoxicity ↑SIRT1-dependent autophagy	**Resveratrol** in mice [110] ↓A53T α-syn-induced neurotoxicity ↓motor and cognitive deficits ↓total α-syn and oligomers, α-syn aggregation ↓neuroinflammation and oxidative stress
***PINK1* mutation** [111]		**Resveratrol** in *PINK1* mutated *Drosophila* [111] ↑lifespan, locomotor activity, and muscle ATP production ↓DA neuron loss and abnormal wing posture ↓mitochondrial aggregates ↑autophagy and mitophagy

Bold: The names of compounds and models.

## Abbreviations

3-MA	3-Methyladenine
6-OHDA	6-Hydroxydopamine
AD	Alzheimer’s Disease
ALS	Amyotrophic Lateral Sclerosis
AMPK	5′ AMP-activated Protein Kinase
Atg	Autophagy-Related-Gene
BAD	Bcl-2-Associated Death Promoter
BDNF	Brain-Derived Neurotrophic Factor
CAT	Catalase
CNS	Central Nervous System
COX-2	Cyclooxygenase-2
Cyt	Cytochrome
DA	Dopamine
DAT	Dopamine Transporter
DLB	Dementia with Lewy Bodies
EGCG	Epigallocatechin Gallate
ER	Endoplasmic Reticulum
ERK	Extracellular Signal–Regulated Kinase
FOXO3	Forkhead Box O3
GDNF	Glial Cell Line-Derived Neurotrophic Factor
GFAP	Glial Fibrillary Acidic Protein
GFP	Green Fluorescent Protein
Gpx	Glutathione Peroxidase
GR	Glutathione Reductase
GSH	Glutathione
GSk3-β	Glycogen Synthase Kinase 3 Beta
GST	Glutathione S-Transferase
HDAC6	Histone Deacetylase 6
HIF-1	Hypoxia-Inducible Factor 1
HMGB1	High Mobility Group Box 1
IFNγ	Interferon Gamma
IIS	Insulin/Insulin-Like Growth Factor Signaling
IL-1β	Interleukine 1 Beta
IL-1β/a	Interleukine-1 beta/alpha
iNOS	inducible Nitric Oxide Synthase
JNK	c-Jun N-Terminal Kinase
LAMP-2A	Lysosomal-Associated Membrane Protein Type 2a
LPS	Lipopolysaccharide
LRRK2	Leucine-Rich Repeat Kinase 2
LSD	Lysosomal Storage Diseases
MALAT1	Metastasis-Associated Lung Adenocarcinoma Transcript 1
MAPK	Mitogen-Activated Protein Kinase
MDA	Malondialdehyde
Meth	Methamphetamine
MMP	Mitochondrial Membrane Potential
MPTP	1-methyl-4-phenyl-1,2,3,6-tetrahydropyridine
MSA	Multisystem Atrophy
mTOR	Mammalian Target of Rapamycin
NADPH	Nicotinamide Adenine Dinucleotide Phosphate Hydrogen
Nf-Kb	Nuclear Factor K Beta
NGF	Neurotrophic Growth Factor
NO	Nitric Oxide
Nrf2	Nuclear Factor Erythroid 2-Related Factor 2
PAF	Pure Autonomic Failure
PARP	Poly (ADP-ribose) Polymerase
PD	Parkinson’s Disease
PGC-1α	Peroxisome Proliferator-Activated Receptor Gamma Coactivator 1-alpha
PINK1	PTEN-induced kinase 1
PKC α	Protein Kinase C alpha
Rab GTPase	Gtp Bound Ras Proteins in Brain
ROS	Reactive Oxygen Species
SDH	Succinate Dehydrogenase
SIRT1	NAD-dependent deacetylase Sirtuin-1
SNARE	Soluble Nsf Attachment Protein Receptor
SNpc	Substantia Nigra Pars Compacta
SOD	Superoxide Dismutase
SQSTM1	Sequestosome-1
SVZ	Subventricular Zone
TBARS	Thiobarbituric Acid Reactive Substance
TFEB	Transcription Factor EB
TGF-b1	Transforming Growth Factor Beta 1
TH	Tyrosine Hydroxylase
TNFα	Tumor Necrosis Factor Alpha
Trk A/B	Tyrosine Receptor Kinase A/B
UCH-LI	Ubiquitin carboxy-terminal hydrolase L1
VEGF	Vascular-Endothelial Growth Factor
VTA	Ventral Tegmental Area
YFP	Yellow Fluorescent Protein
α-syn	alpha-synuclein
